# MicroRNA miR-3646 promotes malignancy of lung adenocarcinoma cells by suppressing sorbin and SH3 domain-containing protein 1 via the c-Jun NH2-terminal kinase signaling pathway

**DOI:** 10.1080/21655979.2022.2036889

**Published:** 2022-02-23

**Authors:** Chun Wang, Bo Cheng

**Affiliations:** a Out-patient Office, The Affiliated Hospital of Jianghan University, Wuhan Sixth Hospital, Wuhan, Hubei, China; b Comprehensive Second Division, The Affiliated Hospital of Jianghan University, Wuhan Sixth Hospital, Wuhan, Hubei, China

**Keywords:** Lung adenocarcinoma, miR-3646, SORBS1

## Abstract

Lung adenocarcinoma (LUAD) is a highly malignant tumor. In this study, we examined the role of miR-3646 and its underlying mechanism in the progression of LUAD. The expression of miR-3646 and sorbin and SH3 domain-containing protein 1 (SORBS1) in LUAD tissues and cells was evaluated by quantitative reverse transcription-polymerase chain reaction. LUAD cell adhesion, proliferation, apoptosis was determined. The targeting relationship between SORBS1 and miR-3646 was verified by dual luciferase and RNA pull-down assays. In vivo assays were performed to verify the in vitro results. The expression of miR-3646 was found to be upregulated in LUAD tissues and cells. MiR-3646 overexpression stimulated the proliferation and adhesion of LUAD cells but inhibite d apoptosis, whereas a miR-3646 inhibitor produced the opposite results. Furthermore, the inhibitory effect of miR-3646 inhibitor was verified in vivo. SORBS1, a target gene identified downstream of miR-3646, was downregulated in LUAD tissues and cells. Additionally, increased SORBS1 inhibited the malignant phenotypes of LUAD cells, which was restored by miR-3646 upregulation. Additionally, western blot analysis revealed that SORBS1 ectopic expression disrupted the JNK signaling pathway, and this effect was restored by miR-3646 overexpression. Thus, this study revealed that miR-3646 promotes LUAD cell proliferation and adhesion, and reduces apoptosis by directly downregulating SORBS1 via the JNK signaling pathway. Investigation of the molecular mechanism of LUAD carcinogenesis revealed that miR-3646 may serve as a biomarker for LUAD treatment.*in vivo*

## Graphical abstract

**Figure:**
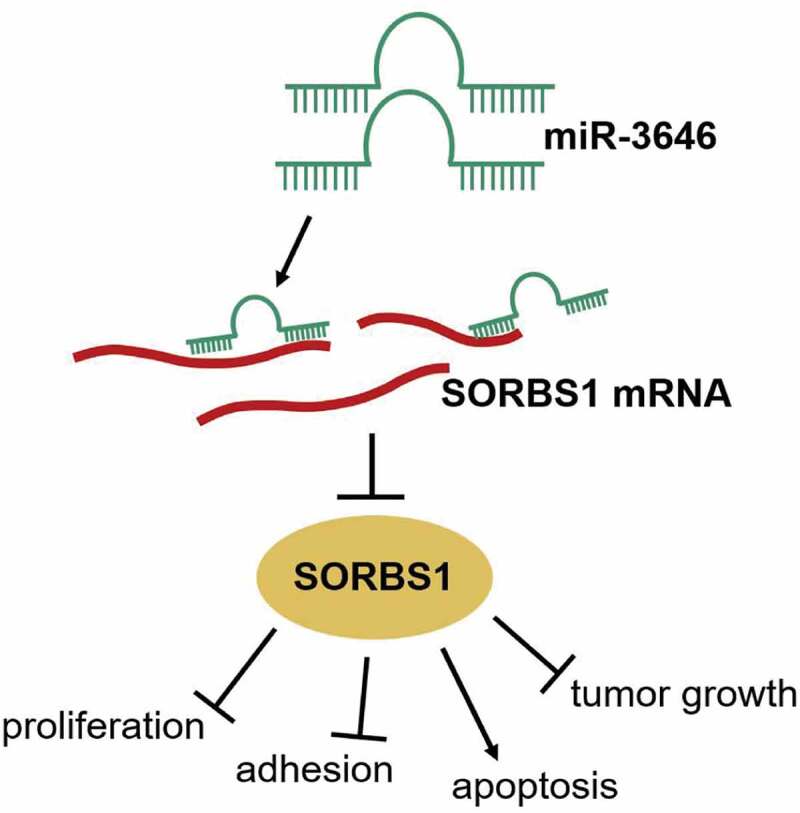

## Background

Lung cancer is a highly malignant tumor. It was estimated that in 2020 the incidence of lung cancer was 2,206,771 (11.4% of all cancer cases), and the number of deaths was 1,796,144 (18.0% of all cancer deaths), contributing to high morbidity and mortality rates worldwide [1]. Although considerable progress has been made in early surgical intervention and chemoprevention of lung cancer, the 5-year survival rate remains low. Because lung adenocarcinoma (LUAD) is often detected at locally advanced and metastatic stages, there are few opportunities for early detection and treatment [2]. Therefore, it is of great significance to develop new treatments and diagnostic methods to effectively improve the prognosis of patients with LUAD. In terms of developing early treatments for LUAD, it is critical to clarify the underlying molecular mechanisms of LUAD occurrence and metastasis.

MicroRNAs (miRNAs) encoded by endogenous genes inhibit target gene translation or mRNA degradation by complementary pairing with target gene, and thereby participate in intracellular biological processes. Previous studies have revealed that miRNAs play crucial roles in suppressing or promoting different cancer processes [3,4]. miR-3646 has been considered to be involved in cancer pathology for over a decade. Previous studies have indicated that miR-3646 acts as a potential oncogene in several cancers, including breast and bladder cancer [5,6]. In addition, upregulation of miR-3646 in breast cancer tissues was found to serve as an indicator of cancer risk [7]. However, the role of miR-3646 in the occurrence of LUAD remains unclear.

Mechanistically, miRNAs modulate mRNA expression by complementary sequence recognition, thereby exerting their functions during cancer malignancy. Bioinformatics analysis predicted sorbin and SH3 domain-containing protein 1 (SORBS1) as a downstream effector of miR-3646. The SORBS1 gene is a coding gene that consists of 36 exons and is located on chromosome 10q24.1. The CBL-related protein encoded by this gene plays a role in insulin signaling and stimulation. There have been limited number of studies on the biological function of SORBS1 in cancer processes in the past two decades. A previous study revealed that SORBS1 is a key regulator of early-to-advanced pathological changes in gastric cancer [8]. In addition, SORBS1 is differentially expressed in different cancers, and this differential expression makes it a biomarker for cancer diagnosis [9,10]. Moreover, SORBS1 mRNA is directly targeted by miRNAs during cancer progression [11]. For example, SORBS1 participates in the osteogenic differentiation of bone marrow mesenchymal stem cells as a putative miR-503-5p target protein [12]. miR-142-5p may regulate the biological behavior of breast cancer cells by targeting SORBS1 [13]. However, the mechanism of action of miR-3646/SORBS1 in LUAD has not yet been reported.

In this study, we aimed to analyze the effect and mechanism of miR-3646 on the malignant behavior of LUAD cells. We hypothesized that miR-3646 enhances cell viability, proliferation, and adhesion of LUAD cells *in vitro*, inhibits apoptosis, and promotes tumor growth *in vivo* by suppressing SORBS1 via the c-Jun NH2 -terminal kinase (JNK) signaling pathway. For the first time, we report the identification of the role of miR-3646 in LUAD progression using bioinformatics analysis and cell functional experiments. A better understanding of the pathogenesis of LUAD mediated by miR-3646 and SORBS1 may provide a theoretical basis for the early treatment of LUAD.

## Methods

### Bioinformatics analysis

GSE130779 [14] and GSE53882 [15], the mRNA and miRNA expression microarrays of LUAD, respectively, were downloaded from the gene expression omnibus (GEO) repository. GEPIA [16] was also used to analyze the mRNA expression in LUAD. The differentially expressed genes (DEGs) were further screened with cutoff of adj P < 0.01 and log fold change (logFC) < −2. Subsequently, the biological processes of the DEGs were analyzed using the Search Tool for the Retrieval of Interacting Genes/Proteins (STRING) database (https://string-db.org/) [17]. The Kaplan-Meier Plotter [18] online prognosis tool was used to identify the key mRNAs that are closely related to the prognosis of patients with LUAD. TargetScan [19] and miRDB [20] were used to predict miRNAs that bind to the gene of interest.

### Clinical tissue acquisition

Overall, 33 pairs of resected LUAD and adjacent normal lung tissue samples were obtained from patients with LUAD who underwent tumor resection surgery at the Ningbo First Hospital between July 2018 and September 2019. The clinical features of these patients are documented in Table 1, and all patients provided written informed consent. The study protocols were approved by the Ethics Committee of Ningbo First Hospital and Medical Clinics.

**Table 1.** Relationship between miR-3646 and SORBS1 and clinicopathological characteristics in 33 cases of LUAD patients

		miR-3646 expression			SORBS1 expression		
characteristics	N = 33	High(N = 17)	Low(N = 16)	P	High(N = 16)	Low(N = 17)	P
Age(years)				0.4384			0.7080
≤60	9	6	3		5	4	
>60	24	11	13		11	13	
Gender				0.1663			0.7319
Male	18	7	11		8	10	
Female	15	10	5		8	7	
Smoking history				0.0136			0.0068
Current	7	1	6		7	0	
Reformed	21	11	10		8	13	
Never	5	5	0		1	4	
Pack years				0.0381			0.0053
≤20	16	5	11		12	4	
>20	17	12	5		4	13	
Tumor stage				0.0167			0.0453
I	5	0	5		4	1	
II	12	5	7		8	4	
III	13	9	4		4	9	
IV	3	3	0		0	3	

### Cell culture

LUAD cell lines (A549, Calu-3, PC9, SPC-A1, and H1975) and a normal lung epithelial cell line (Beas-2B) were provided by BNCC (China). H1975 and SPC-A1 cells were cultured in RPMI-1640 medium containing 10% fetal bovine serum (FBS; Cat#: 16140071, Gibco, USA) and 100 U/mL streptomycin (Cat#: A610494, Sangon, China) under 5% CO_2_ at 37°C. Calu-3 cells were cultured in minimum essential medium (Cat#: E600024, Sangon), whereas A549, PC9, and Beas-2B cells were cultured in Dulbecco’s modified Eagle’s medium high glucose (Cat#: E600003, Sangon) with 5% CO_2_ at 37°C. Additionally, 10% FBS and 100 U/mL streptomycin were added to all media.

### Cell transfection

miR-3646 mimic, miR-3646 inhibitor, miR-3646 negative control (NC), AgomiR-3646, and AgomiR-NC were purchased from Guangzhou RiboBio Co., Ltd. (Guangzhou, China). The pcDNA3.1 plasmid cloned with SORBS1 cDNA (OE-SORBS1) was obtained from GeneCopoeia (Guangzhou, China). Before cell transfection, 6 × 10^5^ PC9 and A549 cells were seeded in six-well plates, and once the cells were approximately 50% confluent, miR-3646 mimic, miR-3646 inhibitor, miR-3646 NC, and miR-3646 mimic plus OE-SORBS1 were transfected into the cells at room temperature using Lipofectamine™ 2000 (Cat#: 11668019; Thermo Fisher Scientific, Waltham, MA, USA), according to the manufacturer’s instructions. After incubating for 2 days at 37°C, the cells were subjected to quantitative reverse transcription-PCR (qRT-PCR) analysis.

### RNA isolation and qRT-PCR

Total RNA was isolated from the acquired samples and harvested cells using TRIzol™ reagent (15596026; Thermo Fisher Scientific Inc.). miRNA was reverse transcribed using the mirVana qRT-PCR miRNA Detection Kit (Cat#: AM1558; Invitrogen; Thermo Fisher Scientific Inc.), according to the manufacturer’s instructions and the expression of miR-3646 was detected. To detect the expression of mRNAs, the isolated RNA was reverse transcribed into cDNA using the SuperScript First-Strand Synthesis SuperMix for qRT-PCR (Cat#: 11752050; Thermo Fisher Scientific, Inc.). Subsequently, the cDNA was subjected to qRT-PCR with TB Green Premix Ex Taq II (Tli RNase H Plus) (Cat#: RR820B, TaKaRa, Japan). The results were obtained using the 2^−ΔΔCt^ method [21]. Glyceraldehyde-3-phosphate dehydrogenase (GAPDH) and Uracil 6 (U6) were used to normalize mRNA and miRNA levels, respectively. The sequences of primers used are listed in Table 2.

**Table 2.** The primer sequences for qRT-PCR

GENE	Primer sequences (5’-3’)
miR-3646	Forward: CCCCAAAATGAAATGAGCC
	Reverse: CAGTGCGTGTCGTGGAGT
U6 SORBS1 GAPDH	Forward: CTCGCTTCGGCAGCACA
	Reverse: AACGCTTCACGAATTTGCGT Forward: CTGTGCCTCGCTCAAAAAGTG Reverse: GGCTCTGCACGATATTTTCTTGT Forward: TGTGGGCATCAATGGATTTGG Reverse: ACACCATGTATTCCGGGTCAAT

### Cell counting kit-8 (CCK-8) assay

The proliferation of LUAD cells was evaluated using the CCK-8 assay. First, 100 μL (containing 2 × 10^3^ cells) of transfected protease-treated suspension of cells in the growth phase was seeded in a 96-well plate and incubated at 37°C for 1, 2, 3, and 4 days. Next, 10 μL of the CCK-8 reagent (Cat#: E606335; Sangon) was added to each well. After incubation with the CCK-8 reagent for 2 h, the absorbance was read at a wavelength of 450 nm [22].

### Bromodeoxyuridine (BrdU) assay

Cell proliferation capacity was assessed using the CytoSelect BrdU Cell Proliferation ELISA Kit (Abcam, USA). First, 100 μL (containing 1 × 10^5^ cells) of trypsin-treated suspension of A549 and PC9 cells was placed in a 96-well plate. Subsequently, 20 μL of diluted 1× BrdU labeling solution was added to the cells, and the cells were cultured for 2 h at 37°C. The cells were fixed with 200 μL of fixing solution at 20°C for 20 min. The cells were then incubated with diluted anti-BrdU monoclonal antibody solution (100 μL) for 1 h and then with diluted peroxidase goat anti-mouse IgG conjugate (100 μL) at 20°C. After 30 min, 100 μL of TMB substrate was added, followed by incubation for 30 min. A microplate reader was used to read the plates at 450 nm [23].

### Cell matrix adhesion assay

First, 40 μg/mL collagen I solution (Cat#: A1048301; Thermo Fisher Scientific Inc.) was prepared in phosphate buffered saline (PBS) and stored at 4°C. A 96-well plate was coated with 40 μg/mL collagen I solution (30 μL/well) at 4°C. After 12 h, the collagen I solution was removed, and the plate was air-dried at room temperature. The transfected A549 and PC9 cells were cultured in serum-free medium for 24 h, and then sufficiently separated with a solution containing 0.05% trypsin and 10 mM EDTA. Next, 100 μL of cell suspension (1 × 10^5^) was added to each collagen I-coated well. The plate was incubated at 37°C for 20 min to allow the cells to attach to the surface. After washing away all non-adherent cells, the adherent cells were incubated at 37°C for 4 h in a medium containing 10% FBS. Subsequently, 10 μL of CCK8 solution was added to each well, and the incubation was continued for another 2 h at 30°C. The absorbance was measured at 450 nm using a spectrophotometer (Thermo Fisher Scientific Inc.) [23].

### Caspase-3 activity assay

Cell apoptosis was detected using the Caspase-3 Colorimetric Assay Kit (Medical and Biological Laboratories, Japan) by measuring the activity of caspase-3. A549 and PC9 cells (2 × 10^5^) were collected and lysed. Next, 50 μL of the cell lysates was placed in each well of 96-well plates, followed by the addition of 50 μL of reaction buffer and 5 μL of caspase-3 substrate. After incubating for 1 h at 37°C, the absorbance was measured at 405 nm using a microplate reader (Bio-Rad, USA). The relative enzymatic activity of caspase-3 compared to that of the blank group was calculated for statistical analysis [24].

### Xenograft assays

The assay was performed according to a previously described protocol [25]. Twelve 4–6-weeks-old BALB/c male nude mice were obtained from the Experimental Animal Center of Wuhan University (Wuhan, China). All mice were maintained at 23 ± 2°C with a 12 h light/12 h dark cycle. The mice had *ad libitum* access to food and water. First, 3 × 10^6^ A549 cells transfected with AgomiR-3646 or AgomiR-NC were subcutaneously injected into the mice. A Vernier caliper was used to measure the tumor size every week. Tumor xenografts were isolated and weighed when the mice were euthanized on day 35.

### Luciferase reporter assay

The luciferase reporter assay was conducted as previously described [26]. Based on the putative miR-3646 binding sites in SORBS1 3ʹUTR predicted by TargetScan, we used psiCHECK-2 to construct four luciferase reporter vectors cloned with wild-type human SORBS1 or mutant human SORBS1 containing mutated sequences in the first, second, or both predicted SORBS1 binding sites involved in interaction with miR-3646. These were named WT, Mut1, Mut2, and Co-Mut constructs, respectively. Next, the WT, Mut1, Mut2, and Co-Mut constructs were introduced into A549 and PC9 cells along with miR-3646 mimics or NC. After culturing for 48 h, the culture medium was collected and used for a luciferase activity assay using a Dual-Luciferase® Reporter Assay System (Promega, USA).

### RNA pull-down assay

The interaction of miR-3646 with its downstream target SORBS1 mRNA was experimentally identified using an RNA pull-down assay. Briefly, 1 × 10^7^ A549 and PC-9 cells were lysed with lysis buffer. Subsequently, the cell lysates were incubated with biotin-labeled miR-3646 probe or biotin-labeled miRNA NC (RiboBio, China) for 2 h at room temperature, followed by 4 h of incubation with streptavidin magnetic beads (RiboBio, China) at room temperature. Next, the RNA bound to the streptavidin magnetic beads was eluted with elution buffer, and the enrichment of SORBS1 in the extracted RNA was finally analyzed by qRT-PCR [27].

### Western blot analysis

To extract total proteins, the transfected cells were lysed with RIPA lysis buffer (Cat#: C500005, Sangon) containing 5 mM EDTA and PMSF. The extracted proteins were subjected to quantitative analysis using the Pierce™ BCA Protein Assay Kit (Thermo Fisher Scientific). Ten micrograms of protein was separated by 10% sodium dodecyl sulfate polyacrylamide gel electrophoresis (SDS-PAGE) and electro-transferred to a polyvinylidene fluoride membrane. The membrane was blocked with 5% skim milk for 2 h at room temperature. Next, the membrane was incubated with diluted primary antibodies against SORBS1 (1:1000, Cat#: PA5-55656, rabbit human; Thermo Fisher Scientific Inc.), Bax (Cat#:ab32503, 1:2000, Abcam), Bcl-2 (Cat#:ab32124, 1:1000, Abcam), GAPDH (1:1000, Cat#: A300-639A, rabbit human; Thermo Fisher Scientific Inc.), p-JNK (Cat#: ab124956, 1:10,000, Abcam), and JNK (Cat#: ab208035, 1:10,000, Abcam) overnight at 4°C. Subsequently, the membrane was incubated with diluted goat anti-rabbit IgG antibody (1:5000, Cat#: A32731, Thermo Fisher Scientific Inc.) at room temperature for 2 h. Finally, the protein bands were visualized using a hypersensitive enhanced chemiluminescence kit (Cat#: C510043, Sangon) [28].

### Statistical analysis

GraphPad Prism 8.0 software (GraphPad, USA) was used to analyze the data. All experiments were repeated three times. One-way or two-way analysis of variance (ANOVA) was applied for data comparison among multiple groups, followed by Dunnett’s or Tukey’s *post hoc* test. For two groups, Student’s *t* test was applied. A P-value < 0.05 was considered to indicate statistical significance, and P-value < 0.001 was regarded as statistically highly significant.

## Results

In this study, we aimed to analyze the effect and mechanism of miR-3646 on the malignant behavior of LUAD cells. We found that miR-3646 levels were upregulated in LUAD tissues and cells, whereas SORBS1 was downregulated. miR-3646 stimulated the proliferation, adhesion, and tumor growth of LUAD cells while inhibiting apoptosis by targeting SORBS1 and activating the JNK signaling pathway. The pathogenesis of LUAD mediated by miR-3646 and SORBS1 may provide a theoretical basis for early treatment of LUAD.

### miR-3646 and SORBS1 identified as probable biomarkers in LUAD

**Figure 1.**
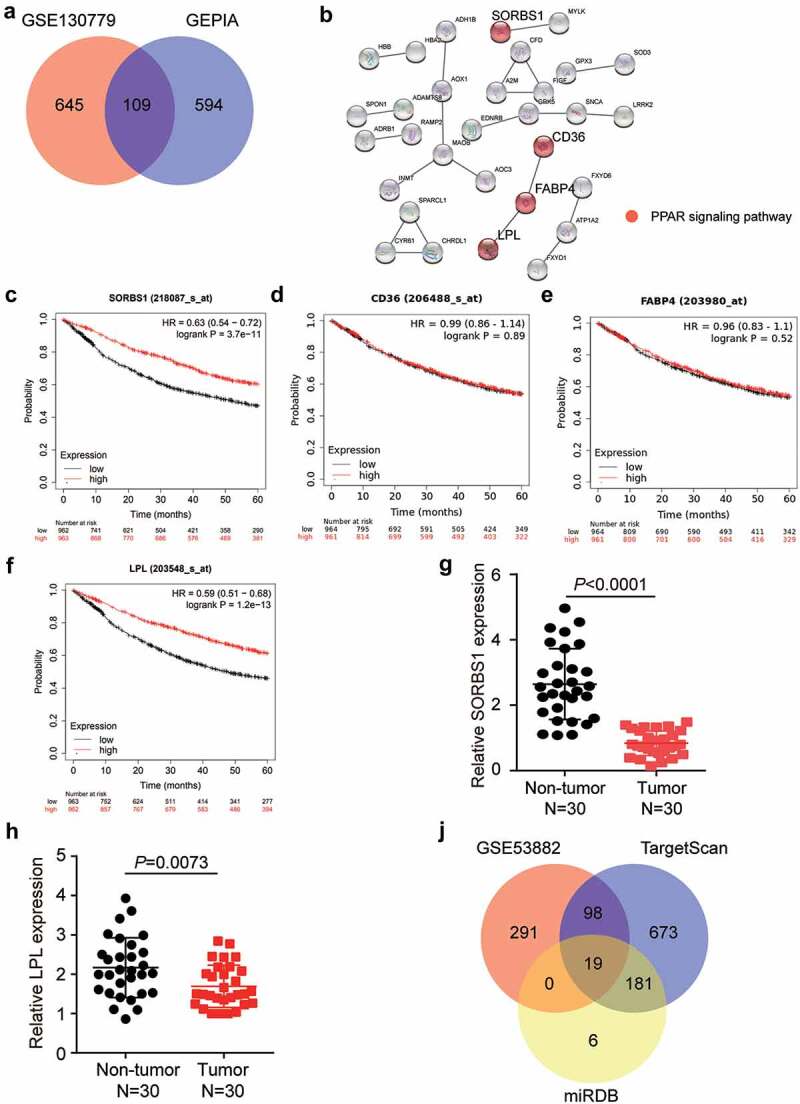
MiR-3646 and SORBS1 were identified as probable biomarkers in LUAD. (a) 109 DEGs were overlapped from GSE130779 and GEPIA with adj.P < 0.01 and logFC<-2. (b) By String analysis, SORBS1, CD36, FABP4 and LPL were found to be associated with the PPAR signaling pathway in LUAD. (c–f) The prognosis analysis found that SORBS1 and LPL was closely associated with prognosis of LUAD. (g–h) The mRNA expression of SORBS1 and LPL in 30 paired tumor and non-tumor tissues. (i) 19 miRNAs were overlapped from GSE53882, TargetScan and miRDB.

By analyzing DEGs from GSE130779 and GEPIA, we identified 109 downregulated DEGs in LUAD with adj P < 0.01 and logFC < −2 (Figure 1(a)). Subsequently, uploading of the 109 DEGs to the STRING database revealed that SORBS1, CD36, FABP4, and LPL were associated with the PPAR signaling pathway (Figure 1(b)). Prognostic analysis of LUAD from the Kaplan-Meier Plotter revealed that low expression of SORBS1 and LPL was associated with a poor prognosis (Figure 1(c–f)). Therefore, we analyzed the expression of SORBS1 and LPL in 30 clinical samples. The results showed that SORBS1 and LPL levels were downregulated in the tumor group compared to that in the non-tumor group (Figure 1(g,h)). Moreover, SORBS1 expression was less than that of LPL in the tumor samples, and thus, SORBS1 was confirmed as the gene of interest (Figure 1(g,h)). Subsequently, TargetScan and miRDB predicted 971 and 206 miRNAs targeting SORBS1, respectively. In addition, 408 upregulated miRNAs were screened from GSE53882 with adj P < 0.01 and logFC > 0. Finally, 19 miRNAs overlapped with GSE53882, TargetScan, and miRDB (Figure 1(i)). Among these 19 targeted miRNAs, miR-3646 had a score of 94 according to the prediction from miRDB, which was the highest target prediction score. A review of previous studies revealed that miR-3646 promotes breast cancer progression [29]; however, its function has not been examined in LUAD. Therefore, miR-3646 was confirmed as an miRNA of interest for investigation in LUAD.

### miR-3646 enhanced the malignant phenotype of LUAD cells

**Figure 2.**
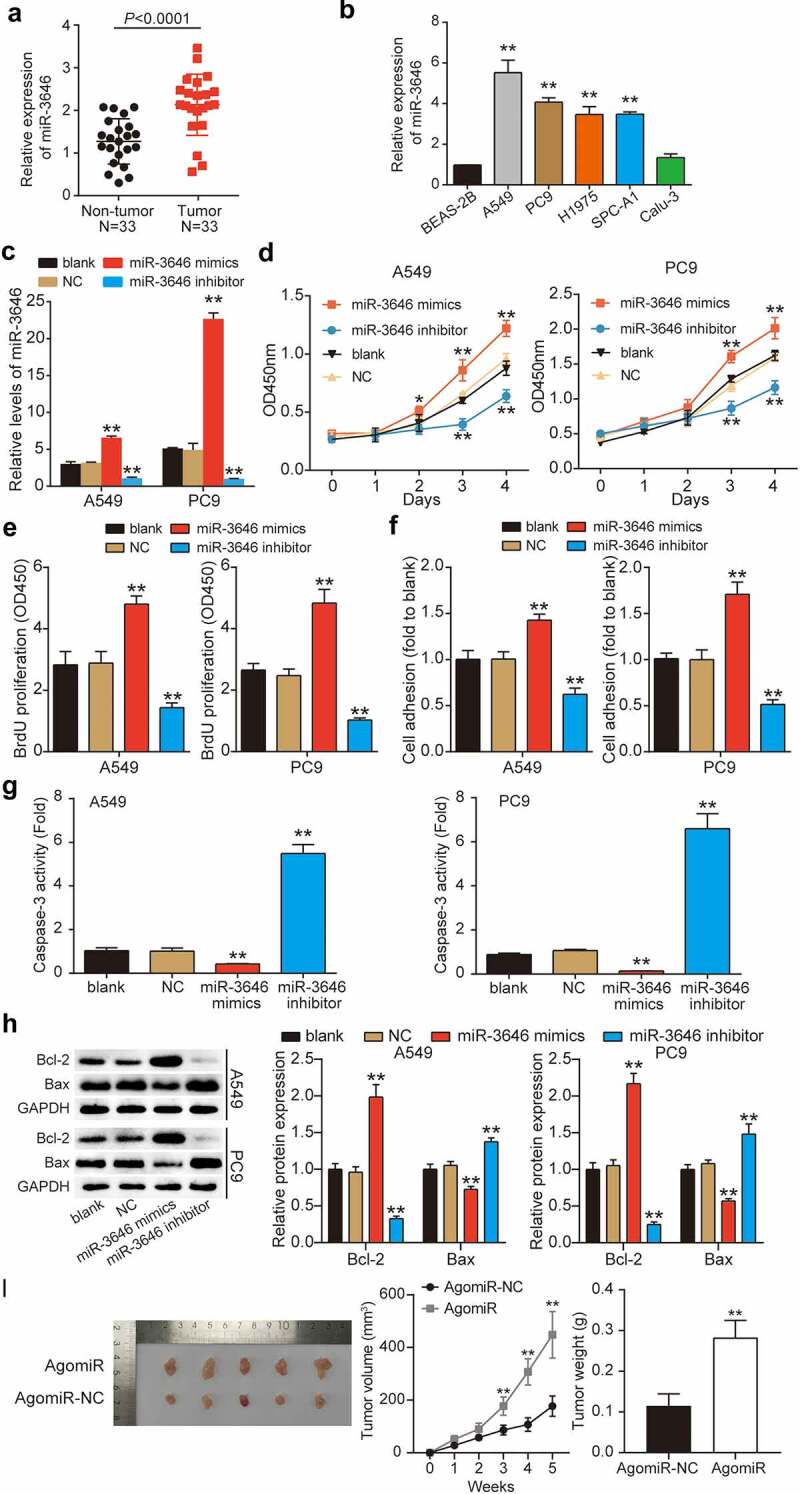
MiR-3646 promoted the malignant phenotype of LUAD cells. (a) MiR-3646 expression in LUAD tissues was determined by qRT-PCR. (b) MiR-3646 expression in LUAD cell lines was analyzed by qRT-PCR. Except for normal lung epithelial (Beas-2B) cell line, the others are LUAD cell lines. LUAD cell lines including A549, PC9, Calu-3, SPC-A1 and H1975 showed higher expression of miR-3646 compared to Beas-2B cell line. **P < 0.001, compared with Beas-2B cell line. (c) Transfection efficiency was verified by qRT-PCR. U6 was used as the internal control. MiR-3646 negative control, miR-3646 mimic or miR-3646 inhibitor were transfected into A549 and PC9 cells. **P < 0.001, compared with blank group (untreated cell) (d) The cell viability of A549 and PC9 cells in different treated groups was analyzed by CCK-8 assay. *P < 0.05, **P < 0.001 compared with blank group. (e) The cell proliferation ability of A549 and PC9 cells in different treated groups was determined by BrdU assay. **P < 0.001 compared with blank group. (f) Cell-matrix adhesion assay was conducted to evaluate the cell adhesion capacity in different treated groups. The relative cell adhesion to blank group was calculated and presented. **P < 0.001 compared with blank group. (g) Caspase-3 activity assay was used to measure the cell apoptosis of A549 and PC9 cells in different treated groups. **P < 0.001 compared with blank group. (h) Western blotting was used to measure Bcl-2 and Bax protein levels of A549 and PC9 cells in different treated groups. **P < 0.001 compared with blank group. The error bars were derived from 3 independent parallel experiments. (i). Nude mice tumorigenicity assay, **P < 0.001 vs. AgomiR-NC.

Using qRT-PCR, we first determined miR-3646 expression in LUAD tissues and cells. As shown in Figure 2(a), miR-3646 was upregulated by approximately two-fold in the LUAD tissue samples (P < 0.0001), in contrast to the adjacent non-tumor tissue samples (Figure 2(a)). Furthermore, we examined the correlation between miR-3646 expression and the clinicopathological features of LUAD. As shown in Table 1, the expression of miR-3646 was associated with tumor stage, smoking history, and pack years, whereas it was not significantly associated with age or sex. In addition, LUAD cell lines, including A549 (P < 0.001), PC9 (P < 0.001), SPC-A1 (P < 0.001), and H1975 (P < 0.001), exhibited markedly higher expression of miR-3646 compared to BEAS-2B, whereas Calu-3 showed no significant difference (P = 0.5388) (Figure 2(b)). Notably, A549 and PC9 cell lines exhibited more than four-fold higher expression of miR-3646 than BEAS-2B in the above experiments and were therefore selected for follow-up experiments. To study the effect of miR-3646 on LUAD cells, we transfected miR-3646 NC, miR-3646 mimic, or miR-3646 inhibitor into A549 and PC9 cells. The qRT-PCR results showed that compared to the blank group, miR-3646 expression level increased significantly in the miR-3646 mimic group (P < 0.001) and decreased in the miR-3646 inhibitor group (P < 0.001), indicating that the cells were successfully transfected (Figure 2(c)). These successfully transfected A549 and PC9 cells were used for subsequent cell phenotype studies. Analysis of the absorption curve of CCK-8 revealed that miR-3646 upregulation effectively increased A549 and PC9 cell viability after 3 and 4 days of culture compared to the blank group (P < 0.001); however, miR-3646 downregulation significantly reduced it (P < 0.001) (Figure 2(d)). Consistent with the CCK-8 assay results, the BrdU incorporation assays revealed that upregulation of miR-3646 evidently stimulated A549 and PC9 cell proliferation, whereas the downregulation of miR-3646 inhibited it compared to the blank group (P < 0.001) (Figure 2(e)). Moreover, the cell matrix adhesion assay revealed that the overexpression of miR-3646 enhanced A549 and PC9 cell adhesion by 1.3-fold, whereas the inhibition of miR-3646 repressed A549 and PC9 cell adhesion by 30% compared to the blank group (Figure 2(f)). In contrast, the results of the caspase-3 activity assay showed that upregulation of miR-3646 suppressed caspase-3 activity by more than 50%, whereas downregulation of miR-3646 enhanced caspase-3 activity by approximately six-fold compared to the blank group (Figure 2(g)). Western blotting was used to evaluate the Bax and Bcl-2 protein levels. The data showed that Bcl-2 protein level was increased and that Bax was reduced in the miR-3646 mimic group compared to the blank group (P < 0.001), whereas the opposite trend was observed in the miR-3646 inhibitor group (P < 0.001) (Figure 2(h)). miR-3646-mediated promotion of cell growth was further confirmed via xenograft experiments, which showed an increase in the size and weight of the transplanted tumors in xenografted mice bearing A549 Agomir-3646 compared to the AgomiR-NC group (Figure 2(i)). Overall, miR-3646 promoted LUAD tumor growth both *in vitro* and *in vivo*.

### miR-3646 reduced SORBS1 expression by binding to its 3ʹUTR

**Figure 3.**
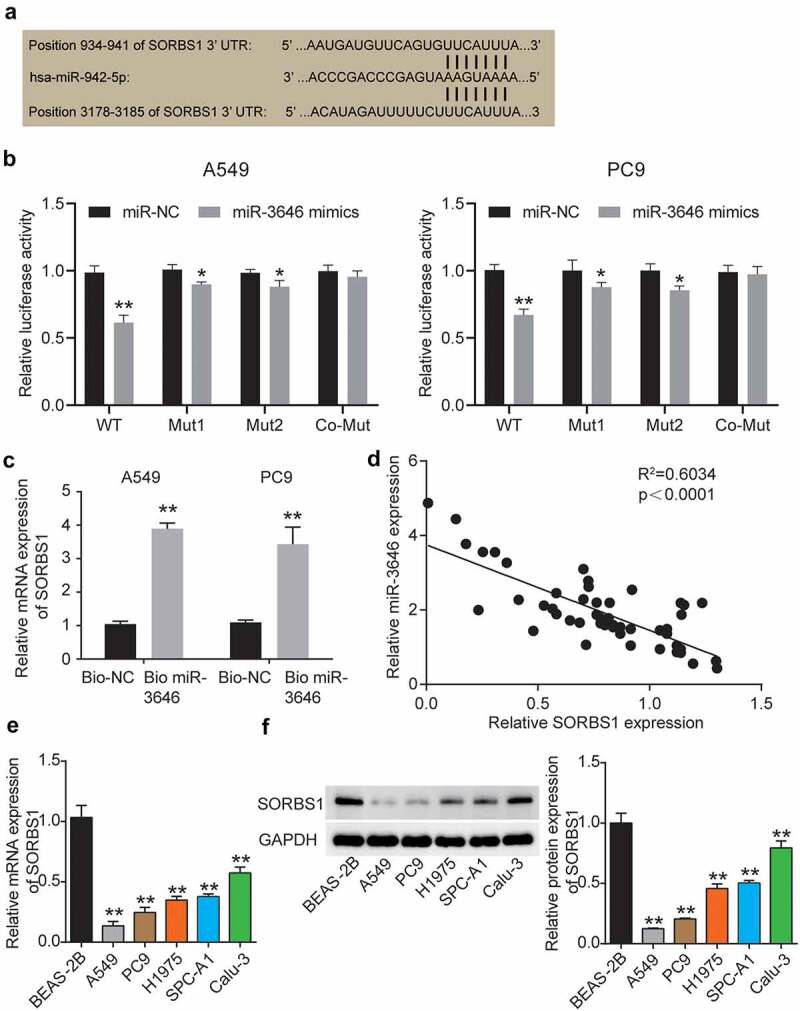
MiR-3646 targeted SORBS1 by binding to its 3ʹUTR oligonucleotide sequence. (a) TargetScan Human 7.2 was used to predict the potential binding site between miR-3646 and its targeted gene. (b) Potential binding between miR-3646 and the 3ʹUTR of SORBS1 mRNA was demonstrated by the luciferase reporter assay. *P < 0.05, **P < 0.001, compared with corresponding miR-NC group. (c) The interaction between SORBS1 and miR-3646 was evaluated by RNA pull-down analysis. **P < 0.001, compared with Bio-NC group. (d) Negative correlation between miR-3646 and SORBS1 expression was identified by Pearson’s correlation analysis. (e) SORBS1 mRNA expression in LUAD cell lines was analyzed by qRT-PCR. Except for Beas-2B cell line, the others are LUAD cell lines. LUAD cell lines including A549, PC9, Calu-3, SPC-A1 and H1975 showed lower expression of SORBS1 mRNA compared to Beas-2B cell line. **P < 0.001, compared with Beas-2B cell line. (f) SORBS1 protein expression in LUAD cell lines was analyzed by Western blotting. Except for Beas-2B cell line, the others are LUAD cell lines. LUAD cell lines including A549, PC9, Calu-3, SPC-A1 and H1975 showed lower expression of SORBS1 protein compared to Beas-2B cell line. The error bars were derived from 3 independent parallel experiments. **P < 0.001, compared with Beas-2B cell line.

To further examine the signaling pathway/s that regulate LUAD pathological processes in downstream of miR-3646 in A549 and PC9 cells, we used TargetScan Human 7.2 to identify the target gene of miR-3646. The program predicted that miR-3646 bound at 934–941 and 3178–3185 sites in the 3ʹUTR of SORBS1 mRNA (Figure 3(a)). We then mutated the oligonucleotide sequence at the binding sites through which miR-3646 binds to the 3ʹUTR of SORBS1 mRNA and constructed fluorescent reporter plasmids SORBS1 mutant 1, SORBS1 mutant 2, and SORBS1 co-mutant. The wild-type and mutant plasmids were transfected into A549 and PC9 cells along with the miR-3646 mimic. As shown in Figure 3(b), the miR-3646 mimic reduced SORBS1 wild-type fluorescence intensity (P < 0.001), but slightly reduced the fluorescence intensity of SORBS1 mutants 1 (A549, P = 0.0416; PC9, P = 0.0191) and 2 (A549, P = 0.0431; PC9, P = 0.0134) compared to the miR-NC + WT group. Notably, the fluorescence intensity of A549 and PC9 cells co-transfected with miR-3646 mimic and SORBS1 co-mutant plasmid (A549, P = 0.9194; PC9, P = 0.9501) was comparable to that of cells co-transfected with NC and SORBS1 co-mutant (Figure 3(b)). These results indicate that miR-3646 directly targets the 3ʹUTR of SORBS1 mRNA. Moreover, the RNA pull-down assay revealed that the SORBS1 level in the Bio-miR-3646 group was enhanced compared to the Bio-NC group, suggesting that the SORBS1 3ʹUTR existed at the binding site for miR-3646 (Figure 3(c)). In addition, Pearson’s correlation analysis revealed a negative correlation between miR-3646 and SORBS1 expression in LUAD tissues (Figure 3(d)). In addition, the correlation between SORBS1 expression and clinicopathological features of LUAD was investigated. The results showed that the expression of SORBS1 was associated with tumor stage, smoking history, and pack years, whereas it was not significantly associated with age or sex (Table 1). Using qRT-PCR and Western blot assays, we determined SORBS1 mRNA and protein expression in LUAD cells. The results showed that SORBS1 mRNA and protein expression was downregulated in LUAD cell lines compared to the normal lung epithelial cell line BEAS-2B (Figure 3(e,f)).

### SORBS1 as a target of miR-3646 suppressed LUAD carcinogenesis

**Figure 4.**
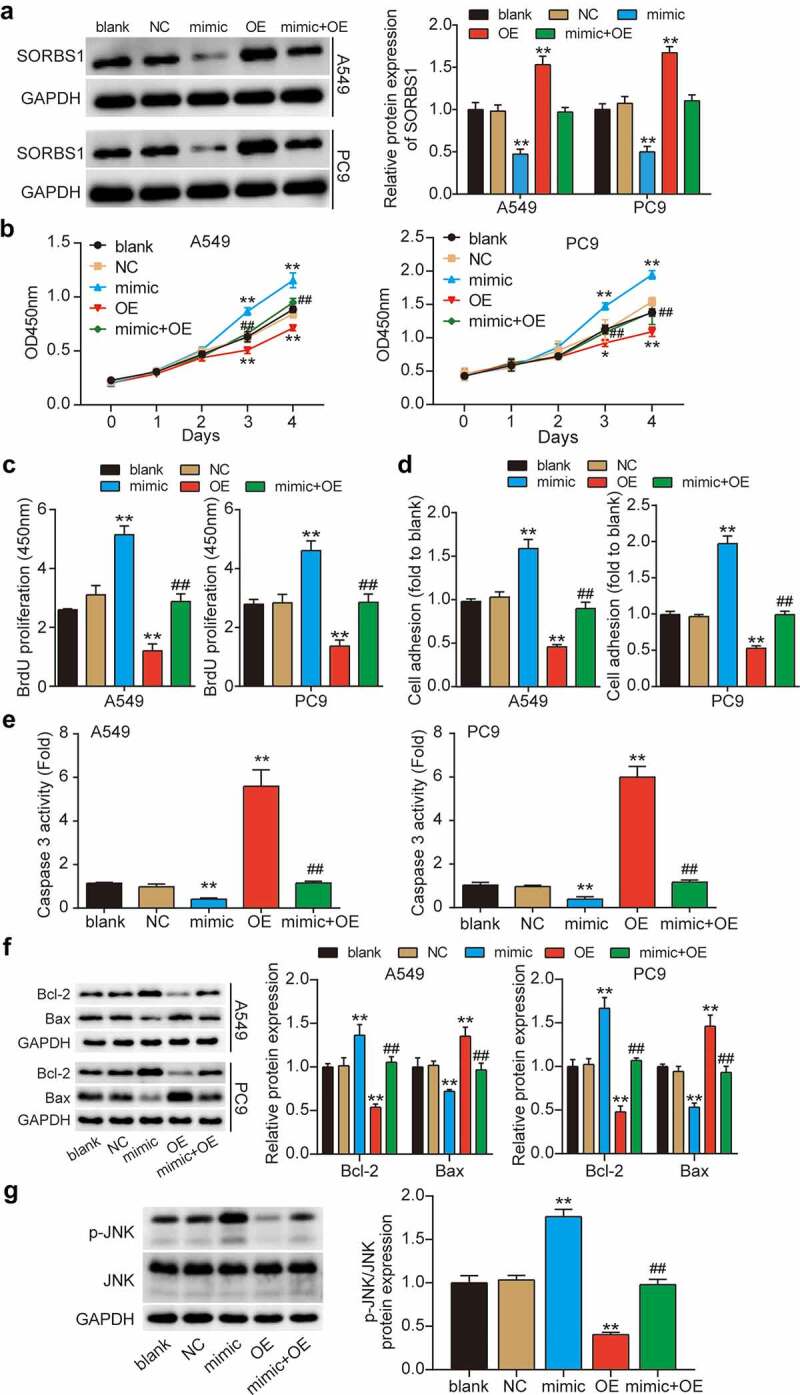
MiR-3646 advanced LUAD by targeting SORBS1 mRNA. (a) Western blot analysis of SORBS1 was used to evaluate the transfection efficiency of A549 and PC9 cells undergoing transfection with miR-3646 mimic (mimic group), OE-SORBS1 (OE), miR-3646 mimic plus OE-SORBS1 (mimic+OE) or negative control (NC), with GADPH as the internal control. Blank group indicated untreated cells. (b) The cell viability of the transfected A549 and PC9 cells was measured by CCK-8 assay. (c) The proliferation of the transfected A549 and PC9 cells was measured by BrdU incorporation assay. (d) Cell-matrix adhesion assay was used to determine the adhesion ability of transfected A549 and PC9 cells. (e) Caspase-3 activity assay was conducted to measure A549 and PC9 cell apoptosis. (f) Western blots analysis of Bcl-2 and Bax protein levels in A549 and PC9 cells. (The error bars were derived from 3 independent parallel experiments). (g) Western blots analysis of p-JNK and JNK. The error bars were derived from 3 independent parallel experiments. *P < 0.05, **P < 0.001, compared with blank group; ##P < 0.001, compared with OE-SORBS1 group.

To further investigate whether SORBS1, which is directly downregulated by miR-3646, inhibits the LUAD process, we transfected OE-SORBS1 (SORBS1 overexpression plasmid), miR-3646 mimic, and miR-3646 mimic plus OE-SORBS1 into A549 and PC9 cells. Western blotting revealed that OE-SORBS1 transfection increased SORBS1 expression by approximately 1.5-fold; however, miR-3646 mimic transfection reduced it by more than 50% compared to the blank group (Figure 4(a)). Furthermore, increased expression of SORBS1 due to OE-SORBS1 in miR-3646 mimic + OE-SORBS1 co-transfected cells was compromised by the miR-3646 mimic. Subsequent CCK-8 and BrdU assays revealed that SORBS1 upregulation decreased the malignant proliferation of LUAD cells compared to the blank group, whereas the miR-3646 mimic restored it (Figure 4(b,c)). Additionally, elevated SORBS1 levels significantly reduced the adhesion of A549 and PC9 cells by approximately 50% compared to the control group, and this reduction was rescued by the downregulation of miR-3646 (Figure 4(d)). In addition, the caspase-3 activity assay (results in Figure 4(e)) revealed that SORBS1 overexpression promoted A549 and PC9 cell apoptosis by approximately 5.5-fold compared to the blank group, and the miR-3646 mimic completely reversed this effect. Moreover, Western blotting showed that Bcl-2 protein level was reduced and that of Bax was increased in the OE-SORBS1 group compared to that in the blank group, whereas the miR-3646 mimic restored it (Figure 4(f)). SORBS1 disrupts the JNK signaling pathway, thereby interfering with breast cancer progression [30]. Hence, we further examined the status of the JNK signaling pathway in A549 and PC9 cells following miR-3646 or SORBS1 overexpression. As illustrated in Figure 4(g), phosphorylation of JNK was noted following miR-3646 overexpression compared to that in the blank group, whereas this phenomenon was reversed by simultaneous overexpression of miR-3646 and SORBS1. These results suggest that miR-3646 sponges SORBS1 and disrupts the JNK signaling pathway, promoting the malignant behavior of LUAD cells.

## Discussion

Using gain-of-function assays our study demonstrates for the first time that miR-3646 is a novel onco-miRNA that mediates LUAD progression by stimulating LUAD cell proliferation and adhesion, and decreasing apoptosis. Specifically, miR-3646 targets SORBS1 3ʹUTR and downregulates SORBS1 expression, resulting in the attenuation of the tumor-suppressive role of SORBS1 during LUAD progression. To the best of our knowledge, for the first time the present study identified miR-3646 as a promising therapeutic target for treatment of LUAD.

To date, several studies have suggested that multiple miRNAs identified as oncogenes in LUAD participate in the progression of cancer [31–33]. For instance, miR-199a-3 inhibits the growth of LUAD cells *in vitro* and *in vivo*, while increasing the apoptosis rate [23]. miR-2355-3p is associated with the diagnosis and prognosis of LUAD and plays a role as an oncogenic factor [34]. The role of miR-3646 in human cancers is limited. For example, it was reported that the overexpression of miR-3646 due to chromosome amplification and increased copy number status might result in it being a potential oncogene in bladder cancer and urothelial carcinogenesis [5]. Moreover, miR-3646 has been identified as a potential oncogene in breast cancer, and increased expression of miR-3646 may represent a new biomarker that could be used to diagnose and predict therapeutic response [6,7]. In addition, miR-3646 was found to promote the malignant phenotype of breast cancer cells by accelerating the cell cycle, whereas knocking down miR-3646 significantly repressed the aggressiveness of the cells [29]. In contrast, miR-3646 has been shown to promote resistance of breast cancer cells to docetaxel [35]. The oncogenic role of miR-3646 has been investigated in several human cancers. However, the role of miR-3646 in LUAD remains unknown. Herein, consistent with the reports on bladder cancer urothelial carcinogenesis and breast cancer, we demonstrated that miR-3646 is abundantly expressed in LUAD tissues. Furthermore, we analyzed the effect of miR-3646 on the malignant behavior of LUAD cells *in vivo* and *in vitro*, and found that miR-3646 overexpression augments LUAD cell proliferation, adhesion, and tumor growth whereas it impairs cell apoptosis. Therefore, consistent with the results of previous studies, this study is the first to confirm that miR-3646 may play a tumor-promoting role in LUAD progression, which broadens the understanding of the molecular targets of LUAD.

A previous study showed that following insulin stimulation in the insulin signaling pathway, SORBS1 mediated the functional transition of the insulin receptor from metabolism to cancer-related cell proliferation as well as migration [36]. The conformational change in SORBS1 following insulin stimulation reportedly causes it to interact with the c-Abl oncoprotein to play a role in the insulin signaling pathway of the human liver cancer cell line Hep3B [37]. Disruption of the JNK signaling pathway resulting from SORBS1 overexpression contributes to decreased breast cancer sensitivity to cisplatin [30]. Moreover, microarrays have shown differential expression of SORBS1 in different cancers, with its upregulation in colorectal cancer and downregulation in prostate cancer [9,10]. This differential expression is considered to be related to the role of SORBS1 in promoting or suppressing cancer. Additionally, bioinformatics analysis has revealed that SORBS1 is involved in the maintenance of adhesion in gastric cancer cells [8]. SORBS1 functions as a metastatic molecule, favoring colorectal cancer cell proliferation, migration, and invasion [38]. In contrast, low expression of SORBS1 is closely correlated with the clinical outcome of patients with breast cancer, and SORBS1 silencing activates oncogenic JNK signaling to promote tumor progression [30]. Therefore, the role of SORBS1 in cancer may be context-dependent. The present results demonstrate that SORBS1 expression is low in LUAD tissues, which is similar to the expression of SORBS1 in breast cancer. In addition, this study is the first to reveal that impairment of proliferation and activation of apoptosis are the consequences of ectopic expression of SORBS1 in LUAD cells, suggesting its tumor-suppressing role during LUAD malignancy. More importantly, miR-3646 overexpression abrogated the effect of SORBS1 overexpression in LUAD cells. The negative correlation between miR-3646 and SORBS1 expression validated the targeting regulation between them. Furthermore, similar to the results reported by Song et al. [30], we also found that SORBS1 overexpression activates the JNK signaling pathway. In addition, based on the targeted regulation of SORBS1 by miR-3646, we further revealed that overexpression of miR-3646 abrogated the role of SORBS1 in interrupting the JNK signaling pathway. These results indicate that miR-3646 recognizes SORBS1 3ʹUTR, resulting in downregulation of SORBS1 expression, activation of the JNK signaling pathway, and promotion of LUAD proliferation *in vitro*, which further clarifies the mechanism of LUAD occurrence and regulation to a certain extent.

In this study, A549 and PC9 were used as model cells to study the malignant behavior of LUAD *in vivo* and *in vitro*, avoiding individual differences caused by internal environmental factors. The use of these cell lines facilitated the study of the functional changes in tumor cells, and enabled the study of the mechanism by which miR-3646 and its target gene SORBS1 participate in the carcinogenesis of LUAD cells at the genetic and molecular levels. In addition, considering the possible changes in cellular processes in an *in vitro* system, we established a xenograft tumor model to investigate the effect of miR-3646 on LUAD cell growth *in vivo*. The results demonstrated that miR-3646 inhibits SORBS1 through the JNK signaling pathway, thus promoting the malignancy of LUAD cells. However, this study has several limitations. For instance, this study only investigated the biological function of LUAD cell lines (A549 and PC9) with high miR-3646 expression, and did not address the influence and mechanism of low expression of miR-3646 in cell lines (Calu-3 and SPC-A1). In addition, a previous study confirmed that SORBS1 forms a complex with AHNAK nucleoprotein (AHNAK), which functions as a tumor suppressor by inhibiting phosphorylated extracellular regulated kinase (ERK) and Rho-associated coiled-coil containing protein kinase 1 [38]; therefore, we speculate that SORBS1 may regulate the development of LUAD by affecting the activities of ERK and Rho. In future studies, we will obtain more clinical samples and select LUAD cell lines with high and low miRNA-3646 expression to perform various cell biological functional experiments to further explore the upstream regulators of miR-3646, the downstream signaling pathway of SORBS1 in LUAD, and the effect of the miR-3646/SORBS1 axis *in vivo*. More importantly, the correlation between miRNA-3646 and SORBS1, and the prognosis of patients with LUAD needs to be further explored.

## Conclusion

In conclusion, we examined the interaction between miR-3646 and its target gene, SORBS1, in LUAD for the first time. We found that miR-3646 was overexpressed in LUAD, whereas SORBS1 was under expressed. miR-3646 promoted the proliferation, adhesion, and tumor growth of LUAD cells, whereas it inhibited apoptosis by targeting SORBS1 mRNA and activating the JNK signaling pathway. To the best of our knowledge, this study furthers our understanding of the pathogenesis of LUAD, and provides valuable biomarkers for the diagnosis of LUAD and promising molecular targets for its clinical treatment. Potent suppression of the miR-3646/SORBS1 axis may be a therapeutic strategy for the management of LUAD.

## Data Availability

The datasets used and/or analyzed during the current study are available from the corresponding author on reasonable request.

## Highlights

- miR-3646 was upregulated in LUAD tissues and cells, whereas SORBS1 was downregulated.
- miR-3646 overexpression stimulated the proliferation, adhesion, and tumor growth of LUAD cells and inhibited apoptosis.
- SORBS1 was identified as a target gene downstream of miR-3646.
- SORBS1 overexpression suppressed the malignant phenotypes of LUAD cells, which was restored by upregulation of miR-3646.
- miR-3646 overexpression activated the JNK signaling pathway in LUAD cells, whereas SORBS1 overexpression inhibited it.
